# Large-scale 3-D interconnected Ni nanotube networks with controlled structural and magnetic properties

**DOI:** 10.1038/s41598-018-32437-8

**Published:** 2018-09-28

**Authors:** Joaquín de la Torre Medina, Tristan da Câmara Santa Clara Gomes, Yenni G. Velázquez Galván, Luc Piraux

**Affiliations:** 1 Instituto de Investigaciones en Materiales/Unidad Morelia, Universidad Nacional Autónoma de México, Morelia, Mexico; 2 Institute of Condensed Matter and Nanosciences, Université catholique de Louvain, Louvain-la-Neuve, Belgium

## Abstract

Large-scale, electrically interconnected three-dimensional (3-D) Ni crossed nanotube networks have been fabricated using an electrochemical dealloying method within the crossed nanopores of polymer host membranes. This method paves the way for the easy and cost-effective fabrication of 3-D magnetic NT networks with precise spatial arrangement and diameter and wall thickness of 10–100 nm controlled individually. The excellent control over geometrical parameters and morphological features of the Ni crossed nanotube networks leads to tunable magnetic and magneto-transport properties. Particularly, the low field magneto-transport behavior is consistent with the expected vortex-like states formed in different segments of the nanotube scaffold, whereas nucleation of domain walls at the intersection of the nanowire segments play a dominant role in the solid crossed nanowire networks counterpart. The present 3-D networks of nanomagnets are of special interest due to their potential for memory devices, computing architectures, sensing and biomedical applications.

## Introduction

In the recent years, the study of magnetic and magneto-transport properties in three-dimensional (3-D) nano-objects made as curved shapes and complex architectures has become the center of an intense research activity^1–3^. Curvature and interconnectivity induced effects in morphologically controlled 3-D systems allow to take advantage of the interaction between vectorial physical parameters like electrical current and magnetization and high frequency signals. For instance, race track memories in 3-D configurations^4,5^, shapeable magnetoelectronics^6,7^, stretchable spin valves^8,9^, flexible anisotropic magnetoresistance (AMR) sensors^10^ and microfluidic-based biomedical applications^11^ are among the device applications that are currently taking advantage of 3-D nanomagnetism. The key aspects of these technologies deal with advanced device size reduction and the possibility of detecting and modulating accurate magnetoresistive signals via the exact 3-D morphology and the stretching state of the materials^12,13^. On the other hand, an interesting kind of complex 3-D nanomaterials recently under study are crossed nanowires (CNW) due to their potential use as 3-D magnetic sensors, robust self-supported microwave devices and logic devices^14–17^. Therefore, understanding the influence of the morphological features of 3-D nano-architectures on their magnetic and magneto-transport properties is of paramount importance for the development of device applications with fine tuning responses. In this sense, magnetic nanotubes (NTs) are interesting cylindrical nanostructures to be arranged in a 3-D morphological architecture because its magnetic and magneto-transport behavior is different to that of solid nanowires (NWs). The hollow core of the NTs provides an additional geometrical degree of freedom that is responsible of modifying magnetization reversal mechanisms and magnetic parameters like the coercive field, remanence squareness and dipolar coupling^18–22^. The ease and agility of the fabrication process of dense interconnected CNW networks by electrochemical deposition motivates its application for the synthesis of another type of 3-D architecture consisting of well-defined crossed nanotube (CNT) networks. The unique features like large-scale electrical interconnectivity and cost-effective fabrication, in contrast to parallel NTs fabricated by other techniques like ALD and CVD, make CNT networks very interesting systems for its use as nanoelement-based macroscopic magnetoresistive devices. In this work, we explore the applicability of the electrochemical dealloying method along with the use of track etched polycarbonate (PC) membranes with interconnected cylindrical nanopores in order to obtain Ni CNT networks. Despite the complex interconnected morphology of the porous membranes, exact inverse replicas made of CNT networks with well defined wall thicknesses are obtained at different reduction potentials. This is the first time to our knowledge that this electrochemical method is used to obtain magnetic interconnected nanotube architectures with predefined sizes and shapes and precise spatial control. Based on simple phenomenological arguments, it is possible to propose and validate an expression that states the linear increase for the wall thickness as a function of the diameter of the nanotubes for a fixed reduction potential. Such a mathematical expression has been proved to be valid not only for the CNT networks studied in this work, but also for any other tubular nanostructures fabricated using the electrochemical dealloying technique. As a consequence, the induced structural and morphological changes in the CNTs result in tunable magnetic properties despite the complex crossed morphology of the networks. Additionally, the differences observed at low magnetic fields in the magneto-transport properties of CNT, with respect to the behavior of CNW networks, are consistent with the expected vortex-like states formed in different segments of the nanotube scaffold, whereas for the interconnected NW architecture nucleation of domain walls at the intersection of the NW segments plays a dominant role.

## Results and Discussion

**Figure 1:**
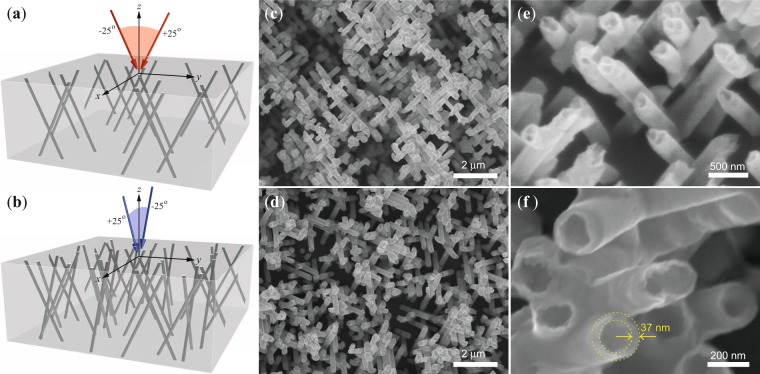
(**a**) Schematics of the first step of the sequential track etching irradiation process, which consists in a first irradiation exposure of the PC film at the two fixed incidence angles of −25° and +25° relative to the OOP direction. (**b**) For the second irradiation step the PC film is rotated in the *x*-*y* plane by 90° and re-exposed to the irradiation flux at the same fixed angles with respect to the OOP direction. SEM images of electrodeposited at a deposition potential of −1 V of (**c**) a Ni CNW network and (**d**) a Ni CNT network obtained after the complete dissolution of the PC nanoporous host template. Close magnification views of the CNT network, showing (**e**) the tubular structure and crossing zones of the nanotubes and (**f**) sharp details of the nanotubes where an average wall thickness of 37 nm has been obtained. The dotted lines are guides for the eye.

In this study, both CNT and CNW networks have been grown in 22 *μ*m thick track etched porous PC membranes with interconnected pores. The morphology of the membranes are defined by exposing a PC film to a first irradiation step at two fixed angles of −25° and +25° with respect to the normal vector to the film plane, as shown in Fig. 1a. After rotating the PC film in the plane by 90°, the second irradiation step takes place at the same fixed angular irradiation flux (see Fig. 1b) to form finally a 3-D nanochannel network. The diameter of the latent tracks are enlarged by following a previously reported protocol in order to obtain 105 nm or 230 nm diameter nanopores (see the METHODS section)^23^. Electrodeposition has been proved to be an excellent tool for filling host porous templates with metallic nanowires with a very high degree of replication of the nanopores. After electrodeposition, the complete dissolution of the PC template leads to an interconnected metallic 3-D self-standing structure, as the one observed for the 230 nm diameter Ni CNW network shown in Fig. 1c. As seen, the CNW network exhibits the replicated complex nanowires branching morphology of the porous template with clear evidence of the fixed polar angle that the nanowires make with the normal to the plane, but also the relative azimuthal orientation angle of 90° between nanowires resulted from the multi-step exposure of the template to energetic heavy ions in the track etching process. These features suggest that the same interconnected morphology must be maintained for the case of CNT networks grown inside the same porous templates. Indeed, a very similar interconnected morphology and self-standing feature is also obtained for CNT networks (see Fig. 1d). Figure 1e clearly shows the tubular structure of the nanotubes along with the fixed orientation between them at the crossing zones. From SEM images, the average wall thickness of the Ni NTs is estimated to be about 37 ± 5 nm, as shown in Fig. 1f. It is worth noting that the observed wrinkles, broken sections and altered morphology of the CNTs is the result of the sudden dissolution of the polymer membrane and the small thickness of the walls of the nanotubes. In contrast, the very low roughness of the PC membrane pores and the strong bond between nanowires and the membrane pores wall^14,24^, suggest that CNT networks embedded into PC membranes have structural features like circular cross sections, low outer surface roughness and non-broken interconnections. These results demonstrate the feasibility of using the electrochemical dealloying method to fabricate large-scale 3-D Ni CNT networks from Cu/Ni - core/shell nanocables. Although the growth of 3-D interconnected conducting polymer nanotubes was recently reported using a similar template-based method^25^, this is the first time to our knowledge that complex networks made of electrically interconnected nanotubes have been fabricated by using a simple, inexpensive and rapid method, which has the potential of allowing for the fine tuning of magnetic and magneto-transport properties. Indeed, previous works on electrodeposited arrays of parallel nanotubes suggest a direct influence of the deposition potential on the nanotube wall thickness and also on their magnetic properties^22,26,27^. In this work, CNT networks were grown at different deposition potentials in order to investigate variations in their magnetic properties as a function of induced changes in the interconnected morphology. Particularly, the magnetic and magneto-transport behavior were shown to depend on the complex hollow geometry of the networks, which have appreciable differences in comparison to its counterpart of solid Ni CNW networks.

**Figure 2:**
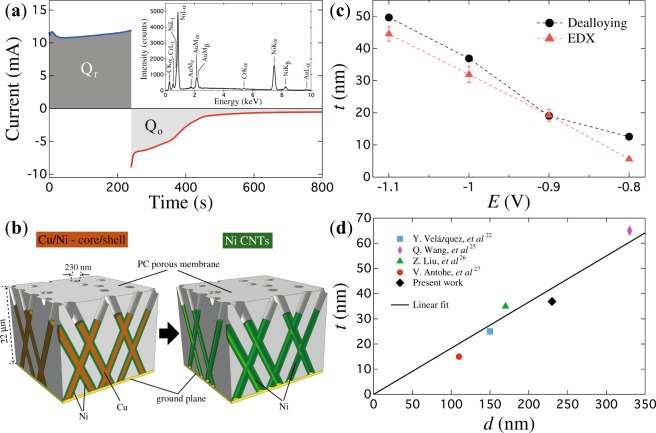
(**a**) Reduction and oxidation curves at the respective potentials of −1.0 V and +0.2 V in order to obtain an initial core(Cu)/shell(Ni) nanocable network and the successive CNT network into a PC membrane with crossed nanopores. The inset shows the EDX spectrum of the CNT network after the complete dealloying process is finished. (**b**) Schematic representations of the cross section of a 3-D crossed core(Cu)/shell(Ni) nanocable network and the successive 3-D CNT network obtained after dealloying of the Cu core. (**c**) Variation of the nanotubes wall thickness *t* as a function of the reduction potential *E* obtained using equation (1) with: *r* given in terms of the reduction and oxidation charges, which are computed as the integral area beneath the corresponding electrochemical curves (circles) and; with *r* given in terms of the core and shell volumes, which correspond respectively to the atomic % of Cu and Ni obtained from EDX mappings (triangles). The error bars for the *t* values obtained from EDX mappings correspond to the standard deviation from the statistical mean value. (**d**) Plot of *t* vs. *d* for the CNT network at *E* = −1 V (lozenge) obtained with equation (1), which is compared to the values reported in the different previous works on parallel NT networks^22,26–28^. The linear regression is carried out to the whole set of *t* vs *d* values with a fitting accuracy of 96%.

In one hand, clear morphological changes of the nanotubes are evident from the electrochemical reduction and oxidation curves for the different deposition potentials, because their corresponding charges depend on the deposition potential. The reduction curve displayed in Fig. 2a, for a specific potential (*E* = −1.0 V), corresponds to the typical electrochemical Ni and Cu co-deposition behavior of a chronoamperometric process. This process leads to a Cu/Ni - core/shell structure as the one displayed schematically in Fig. 2b, which shows the phase separation between the two metallic elements. That is, the Cu reduction at lower potentials facilitates the formation of small Cu islands at the center of the initial growth, whereas the Ni tends to grow at the islands periphery as the solution is depleted from Cu cations^27^. Next, the Cu cores in the core/shell interconnected network are electrochemically etched by applying an oxidation potential of +0.2 V. The corresponding electrochemical oxidation curve is displayed in Fig. 2a at time values just after finishing the electrochemical reduction stage. For this curve, the observed progressive decrease towards zero of the negative electrical current is the consequence of the decrease of the amount of Cu until its complete dissolution is achieved. Indeed, as seen in the inset of Fig. 2a, no remaining Cu is shown in the EDX spectrum recorded for the CNT network of this figure. The principal peaks correspond to Ni, Au and Cr emission lines arising from the Ni CNT network structure and from the Au/Cr cathode of the porous membrane used for the growth of the NTs. This process results in a Ni shell with insignificant etching because of its surface passivation when using sulfamate based electrolytes^27^. Therefore, the obtained 3-D architecture is a CNT network as the one shown schematically in Fig. 2b, as also corroborated by the SEM micrograph shown in Fig. 1d after dissolution of the host PC membrane. Besides, since the reduction or deposition potential (*E*) has a direct influence on the NTs wall thickness, differences in the reduction (*Q*_r_) and oxidation (*Q*_o_) charges are expected for different *E* values. Considering the same Faradaic efficiency for the reduction and oxidation stages in the CNT networks fabrication process and the proportionality between the reduction (oxidation) charge and the core + shell (core) volume, the NTs wall thickness (*t*) can be written in terms of either the volumes or charges ratios *r* = *V*_Cu_/(*V*_Cu_ + *V*_Ni_) = |*Q*_o_|/|*Q*_r_| and the outer NTs diameter *d* as

1 $$t=\frac{1}{2}\,(1-\sqrt{r})\,d.$$

Using equation (1) along with the *Q*_r_ and *Q*_o_ values obtained by numerical integration of the reduction and oxidation curves, as seen by the shaded areas in Fig. 2a, give approximate NT wall thickness values at different deposition potentials *E*. Particularly, a *t* value of 36.9 nm is obtained using equation (1) for the particular case *E* = −1 V, which is in remarkable agreement with the NTs wall thickness value obtained from high resolution SEM images (see Fig. 1d). As seen in Fig. 2c, thicker NT walls are obtained for larger reduction potentials (circles), where this tendency is in good agreement with that reported previously^22,26–28^. Besides, carrying out EDX mappings on various regions on Cu/Ni core/shell CNW networks allow to obtain an statistical average of the atomic % of Cu and Ni which are proportional to the respective material volume. Using Eq. (1) with *r* = *V*_Cu_/(*V*_Cu_ + *V*_Ni_), along with the Ni to Cu volume ratio obtained from the EDX mappings leads to *t* values that are in very good agreement with those obtained from the electrochemical dealloying procedure, as shown in Fig. 2c. As seen, a very good agreement has been found from the comparison between the Ni shell/wall thickness obtained from both experimental procedures. This result validates in one hand, the excellent Ni and Cu phases separation during the electrochemical reduction stage and on the other hand, the replicated cylindrical geometry of the nanowires and nanotubes of the membrane pores. Previous works have also reported different *t* values for NTs of different outer diameters (*d*) in the range from 110 nm to 330 nm, but made at the same reduction potential (*E* = −1 V). Plotting the values of *t* vs *d* from these different previous works and comparing them to the present result for the CNT network fabricated at *E* = −1 V, shows a clear linear relationship between *t* and *d*, and the approximate phenomenological expression *t* ≈ 0.183 × *d* is obtained for this reduction potential, as shown in Fig. 2d. Although a complete understanding of the growth mechanism for electrodeposited Ni-Cu alloy NWs remains elusive, the miscibility gap was proposed to explain phase separation, leading to the characteristic core-shell structure^27,29^.

**Figure 3:**
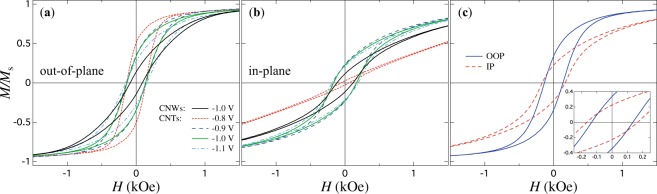
(**a**) Hysteresis loops measured with the magnetic field applied along the out-of-plane (OOP) direction of the PC template for CNT networks (*ϕ* = 230 nm) grown at reduction potentials of −1.1, −1.0, −0.9 and −0.8 V and Cu oxidation potential of 0.2 V. The hysteresis loop for a Ni CNW network with *ϕ* = 230 nm (black continuous line) electrodeposited at −1.0 V is added for comparison. (**b**) Corresponding hysteresis loops for the samples in (**a**) measured with the magnetic field applied along the in-plane (IP) direction of the PC template. (**c**) Comparison between the hysteresis loops measured in the OOP and IP directions for the CNT network fabricated at a deposition potential of −1 V. The inset shows a close view of the same figure at low field values.

On the other hand, the magnetic behavior is analyzed from recorded hysteresis loops at room temperature with the external field applied in the IP and OOP directions. Figure 3a shows hysteresis loops with the applied field in the OOP direction for four Ni CNT networks grown at deposition potentials of −0.8, −0.9, −1.0, −1.1 V. The observed changes in the shape of the hysteresis loops are in good agreement with previous works on arrays of parallel Ni nanotubes^22,26^, suggesting a close relation with variations in the nanotubes wall thickness. By comparing the hysteresis loops in Fig. 3a,b for reduction potentials between −0.9 V and −1.1 V, a systematic change in remanence magnetization is observed. The decrease of this parameter for higher *E* values is attributed to the decrease of the effective magnetic anisotropy as a result of the increase of the NTs wall thickness. In other words, for thicker NTs walls, larger dipolar fields are originated by increasingly closer magnetic charges at the NTs inner surface, whereas lower demagnetizing fields are originated by increasingly more distant magnetic charges in between the NTs inner and outer surfaces. The competition between these fields leads to lower effective magnetic anisotropies that are accompanied by lower remanence magnetization values. As seen in Fig. 3a,b this effect is more important along the OOP direction. This feature is the result of the more rapid increase with *E* of the dipolar interaction to demagnetizing field ratio in the OOP direction than that in the IP direction. Furthermore, as seen in Fig. 3c the hysteresis loops for |*E*| > 0.8 V measured along the IP direction display higher coercive field values than those measured in the OOP direction. This difference can be explained in the light of the dependence of the coercive field, via its corresponding magnetization reversal mechanism, on the relative angle between the applied field and the NTs axis^20^. Indeed, it was shown that when the field angle is small, the vortex reversal mode is the most favored while at larger field angles, a transverse reversal mode is favored^20^. The lower (higher) coercive fields for the OOP (IP) direction confirm a vortex-like (transverse-like) reversal mode at a relative angle of 25° (65°) between the applied field and the NTs axis. On the other hand, for the case *E* = −0.8 V the magnetic behavior in the OOP direction is consistent with the systematic change in shape of the hysteresis loops in Fig. 3a. As expected, the remanence increases and the coercive field has almost the same value as those for the other samples. However, as seen in Fig. 3b, the hysteresis loop measured along the IP direction shows a completely different behavior, as both remanence and coercive field are very close to zero. These features are characteristic of very thin unidimensional nanostructures measured with the magnetic field applied in the direction perpendicular to their axis where the coherent rotation mode is the dominant magnetization reversal mechanism^30^. Therefore, for this configuration the magnetic behavior of CNT networks with very thin NTs walls is consistent with this scenario, since the preferential alignment of the magnetization is along their axis and the magnetic field is applied close to the direction perpendicular to the NTs axis.

As shown hereafter, these changes are expected to have a direct impact on the magneto-transport properties during magnetization reversal measured in both IP and OOP directions. Besides, thicker wall thicknesses are expected at higher deposition potentials up to −1.1V because of the preferential deposition of Ni in contrast to Cu^26^. Although the granular feature of nanotubes makes difficult to elucidate the exact wall thickness from SEM analysis^31^, the magnetic behavior shown in Fig. 3 is consistent with thinner average wall thicknesses for lower deposition potentials. Indeed, the progressive increase of the remanence magnetization for less negative deposition potentials means that the nanotubes magnetization tends to align along their axis, which is consistent with the reduction of the magnetostatic (MS) effects resulting from the smaller effective packing fraction of the CNT networks^21,22,32^. For arrays of parallel NWs, it was accepted that the magnetization reversal is accomplished by nucleation process in small activated volume at the NWs ends and subsequent propagation. In addition, the experimental results have shown that the angular dependence of the coercive field can be described satisfactorily by curling mode^33–36^. For the crossed NW system, the magnetization reversal process is mostly governed by the presence of magnetic domain walls located at the junctions formed by the intersection of the numerous NW segments^4,37^. As shown hereafter, AMR measurements performed on the Ni CNW system exhibit features that may be associated to the presence of domain walls and their propagation through such complex 3-D networks of NWs.

**Figure 4:**
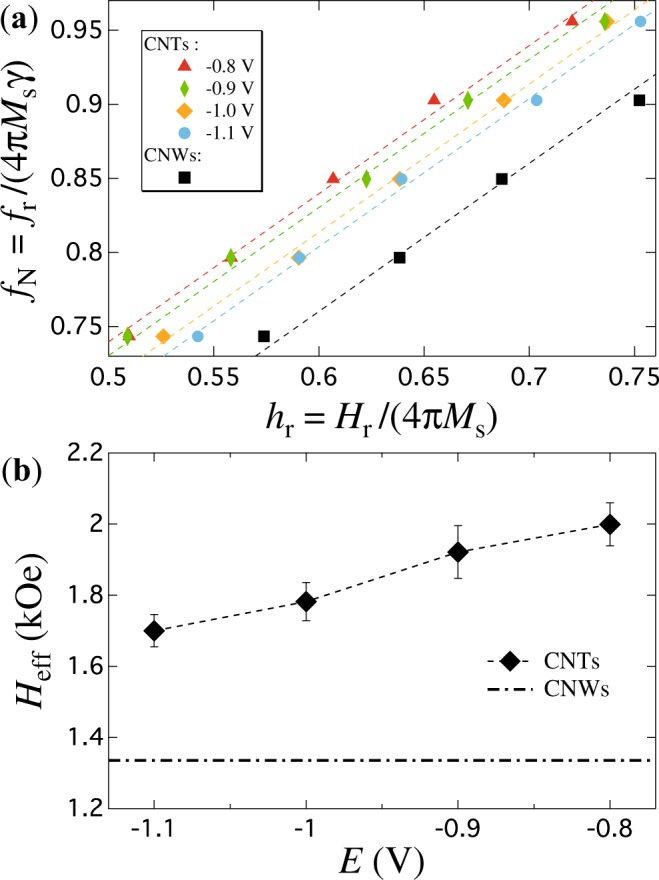
(**a**) Normalized dispersion relations, by the anisotropy field (*H*_F_ = 4*πM*_s_) and zero-field resonance frequency (*γH*_F_) of a continuous thin Ni film, for Ni CNT networks with *ϕ* = 230 nm fabricated at reduction potentials in the range from −0.8 V to −1.1 V. (**b**) Variation of *H*_eff_ as a function of the reduction potential (lozenges) for Ni CNT networks along with the value for a Ni CNW network (dash-dotted line). Error bars are estimated from the standard deviation of the experimental *f*_N_ vs *h*_r_ dispersion relations.

On the other hand, information about the magnetic properties of the distinct CNT networks disregarding magnetization reversal processes can be readily obtained from ferromagnetic resonance experiments, as they are carried out in the saturated state. In the present work, only magnetostatic contributions are considered because of the polycrystalline nature of the as-obtained nanotubes using the deposition/dealloying method^31^. This means that the magnetic anisotropy is controlled only by changes in the dipolar coupling of the networks via variations of the nanotubes wall thickness. Figure 4a shows the FMR dispersion relations for CNT networks with different average wall thicknesses. The data were normalized by the anisotropy field of a continuous magnetic thin film. The advantage of using the normalized dispersion relations lies in the fact that they allow to disconnect distinct magnetic anisotropy contributions^15^, as well as to distinguish fine differences originated by morphological aspects of the networks. As seen, the dispersion relation for the less negative deposition potential (−0.8 V) is shifted upwards with respect to the one fabricated at the more negative potential (−1.1 V). This feature is consistent with higher anisotropy fields with preferential orientation along the OOP direction for less negative potentials, which is due to the fact that thinner NT walls account for lower dipolar interaction fields in the networks^22^. Conversely, the dispersion relation for the CNW network displayed in Fig. 4a corresponds to the lower bond of the allowed resonance frequencies for CNT networks grown in the same porous template. This network represents the limiting case of a non-hollow structure for which a larger competition between the demagnetizing and dipolar fields takes place. Further quantitative analysis taking advantage of the corresponding FMR condition is needed in order to elucidate the influence of geometrical aspects of CNT networks on its effective anisotropy field (*H*_eff_). The basic framework of CNT and CNW networks is such that it replicates the porous architecture of the template, so it consists in ensembles of NTs and NWs spaning along both the *x* and *y* directions with fixed polar orientation angle *θ*_0_ measured with respect to the normal of the porous membrane plane or *z*-axis (OOP: out-of-plane). In this way, interconnections between NWs or NTs take place randomly which give rise to CNT and CNW networks with different morphologies since the former is a hollow structure, whereas the later it is not. However, both networks are made of high aspect ratio cylindrical nanoelements, so the same FMR condition can be used despite their morphological differences. Considering the normalized resonance frequency and resonance field, defined respectively as *f*_N_ = *f*_r_/(*γH*_F_) and *h*_N_ = *H*_r_/*H*_F_, with *γ* = 3.09 GHz/kOe the gyromagnetic ratio for Ni and *H*_r_ the resonance field, the resonance condition for both CNT and CNW networks writes

2 $${f}_{{\rm{N}}}=\sqrt{{h}_{{\rm{N}}}^{2}+{A}_{1}{N}_{{\rm{M}}}{h}_{{\rm{N}}}+{A}_{2}{N}_{{\rm{M}}}^{2}},$$

where *N*_M_ = *H*_eff_/*H*_F_ is the magnetostatic factor of the network, with *H*_eff_ = 4*πM*_s_*N*_M_ and *H*_F_ = 4*πM*_*s*_ the respective effective anisotropy fields for the network and for the continuous magnetic thin film. Equation (2) has been obtained from the Smit and Beljers formalism^38^ (see the Supplementary information), which can be used along with the experimental data to obtain *N*_M_ as fitting parameter. The constants *A*_1_ and *A*_2_ have respective values of 1.464 and 0.528 for the particular case where the orientation angle *θ*_0_ of both CNTs and CNWs is equal to 25°. Notice that *N*_M_ reproduces only the morphological aspects of purely magnetostatic planar networks since it is independent on *M*_s_, so the normalized resonance condition of equation (2) permits to identify precise differences in the morphology of CNW and CNT networks grown under different conditions. Using equation (2) as fitting function to the experimental data, shown as dotted lines in Fig. 4a, the magnetostatic factor and then the effective anisotropy field for the different CNT and CNW networks have been determined. Specifically, Fig. 4b shows a monotonous decrease of *H*_eff_ as the deposition potential increases towards more negative values. The observed trend of *H*_eff_ suggests that increasing the deposition potential further favors the deposition of Ni material with respect to Cu up to the limit where no hollow core in the NTs can be allowed. This limit corresponds to the CNW network embedded in the same porous template, whose *H*_eff_ field represents the lower bond for the expected values for CNT networks (see the dash-dotted line in Fig. 4b). This lower bond is the consequence of the larger competition between the demagnetizing and the dipolar interaction field between NWs, which result from their cylindrical surface charges. In the case of CNT networks, additional surface charges appear at the inner hollow core of the NTs, which lead to a decrease of the dipolar field and then to an increase of *H*_eff_. Indeed, it has been shown that the dipolar field depends on the inner to outer radii ratio *β* = *r*_i_/*r*_e_^22^, such that it tends to zero as *r*_i_ tends to the value of *r*_e_. Conversely, the dipolar field tends to the maximum value for an array of NWs, as the hollow core disappears, that is, as *r*_i_ tends to zero. This behavior fairly explains the variation of the effective field with the NTs wall thickness, as observed in Fig. 4b. These results are in good agreement with the observed behavior shown in Fig. 3 and they help explaining the variation of the remanence magnetization as the potential is modified. However, as mentioned previously, the overall magnetic behavior in unsaturated states must be accompanied by magnetization reversal processes that have a direct influence on the effective anisotropy.

**Figure 5:**
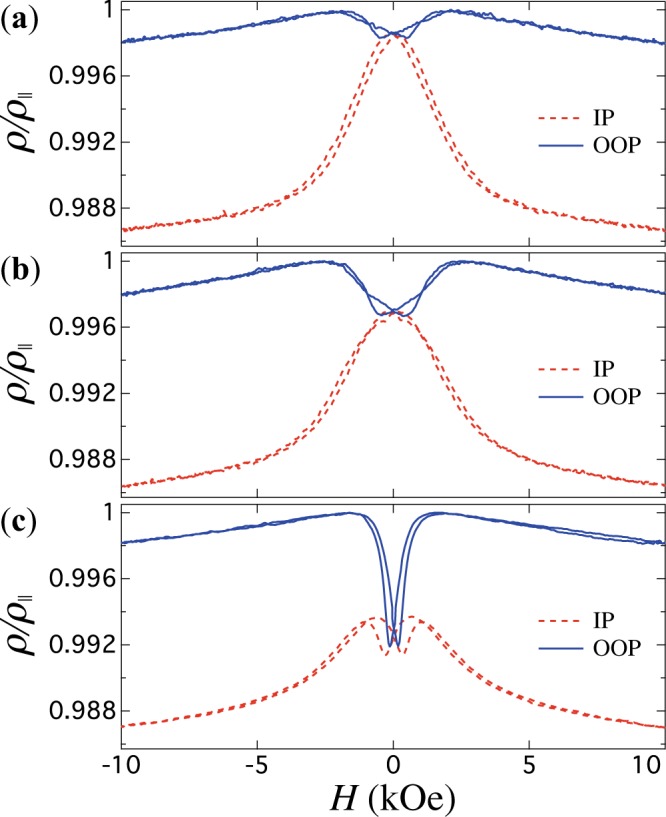
Anisotropic magnetoresistance curves measured with the external field applied in the OOP (continuous lines) and IP (dashed lines) directions to the plane of the porous template for two CNW networks with NWs diameter of (**a**) 105 nm, (**b**) 230 nm and (**c**) for a CNT network with NTs outer diameter of 230 nm.

Besides, magneto-transport measurements constitute another way to investigate the magnetic behavior of CNT networks. Anisotropic magnetoresistance (AMR) measurements, which lead to changes in the resistivity as the relative orientation between the magnetization (*M*) and current (*I*) is modified, is the ratio given by the electrical resistance increase between resistance states when the magnetization is parallel (${\rho }_{\parallel }$) and perpendicular (${\rho }_{\perp }$) to the current. This property along with the shape of the MR curves have been extensively used to identify micromagnetic configurations, magnetization reversal processes and resistance states in arrays of parallel NWs^35,39,40^, individual NWs and NTs^41,42^, nano-networks^43,44^ and complex 3-D CNW networks^15,16^. In this work, AMR is used to obtain a better insight of the magnetic behavior of 3-D CNT networks and its key difference with 3-D CNW networks. Figure 5 shows measured AMR curves for two CNW networks with NW diameters of (**a**) 105 nm and (**b**) 230 nm and (**c**) for a CNT network with NTs diameter of 230 nm (*E* = −1 V). Measurements were carried out with the external field applied along the IP (dashed lines) and OOP (continuous lines) directions. From these measurements, the AMR ratio can be obtained using the known expression^45^

3 $$\frac{{\rm{\Delta }}\rho }{\rho }=\frac{{\rho }_{\parallel }-{\rho }_{\perp }}{{\rho }_{{\rm{av}}}},$$

where ${\rho }_{{\rm{av}}}=\frac{1}{3}{\rho }_{\parallel }+\frac{2}{3}{\rho }_{\perp }$ is the average magnetoresistance in 3-D systems and ${\rho }_{\parallel }={\rho }_{{\rm{ip}}}+{\rho }_{{\rm{oop}}}-{\rho }_{\perp }$ and ${\rho }_{\perp }=[k{\rho }_{{\rm{ip}}}-(1-k){\rho }_{{\rm{oop}}}]/[2k-1]$ are as-obtained expressions for the parallel and perpendicular resistance states for the CNT and CNW networks studied in this work (see the Supplementary information). In these expressions, *k* = 0.8214 and *ρ*_ip_ and *ρ*_oop_ correspond to the measured IP and OOP resistance states at saturation. In view of the tilted angle of the CNWs and CNTs with respect to the normal to the template, the ${\rho }_{\perp }$ values are lower than those for the IP resistance state at saturation (see Fig. 5). This means that the ${\rho }_{\perp }$ state can not be reached directly in the measured AMR curves, so they must be determined using the expression given above. Then, using equation (3) along with the ${\rho }_{\perp }/{\rho }_{\parallel }$ values in Table 1, allows obtaining accurate AMR ratios (see Table 1) for the Ni CNW and CNT networks in Fig. 5. As expected, these values are very close to each other because the AMR ratio is an intrinsic property of magnetic materials. Moreover, these values are in good agreement with previously reported AMR ratios in arrays of parallel NWs^35^. However, quite different magnetoresistive behavior between CNWs and CNTs appears at low magnetic fields, as discussed hereafter.

**Table 1:** Values of the coercive field in the OOP direction, the resistance state ${\rho }_{\perp }$/${\rho }_{\parallel }$ determined using equation (9) and the respective AMR ratio (Δ*ρ*/*ρ*) for CNW networks with NW diameters of 105 nm and 230 nm and for a CNT network with NT diameter of 230 nm.

Sample	${{\boldsymbol{H}}}_{{\bf{c}}}^{({\bf{o}}{\bf{o}}{\bf{p}})}({\boldsymbol{Oe}})$	${{\boldsymbol{\rho }}}_{{\boldsymbol{\perp }}}$/${{\boldsymbol{\rho }}}_{{\boldsymbol{\parallel }}}$	AMR (%)
CNWs (105 nm)	321.5	0.9824	1.78
CNWs (230 nm)	88.2	0.9819	1.83
CNTs (230 nm)	144.3	0.9826	1.76

**Figure 6:**
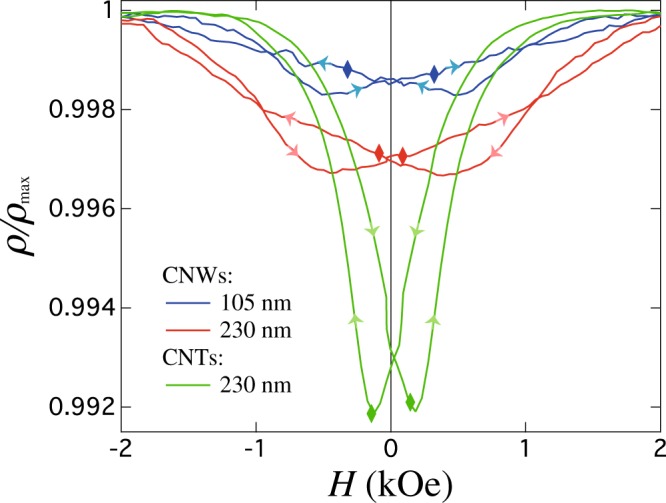
AMR curves recorded in the OOP direction for CNW networks with NW diameters of 105 nm and 230 nm and for a CNT network with outer diameter of 230 nm. The arrows indicate the magnetic field sweep direction from positive to negative or from negative to positive. The lozenges indicate the resistance states at the corresponding coercive field for each network.

We now turn to the discussion of magnetic states observed in Ni CNTs with the magnetic field applied in the OOP and IP directions. The observed coincidence between the AMR curve minimum in the OOP configuration (see Fig. 6) and the resistance at coercivity for the CNT network means that the total local magnetization within NT segments is close to zero, which in this case coincides with the zero effective magnetization of the entire CNT network observed at the coercive field in the hysteresis loop. According to previous works, when the external field is applied at low applied field angles with respect to the NTs axis, the magnetization reversal of NTs is dominated by the curling (vortex) reversal mode where magnetic moments rotate progressively via propagation of a large number of vortex domain walls^18–20^. Since the AMR curve minimum shown in Fig. 6 lies almost at the middle point between the IP and OOP curves, the micromagnetic configuration of the CNTs during magnetization reversal account for a large portion of the moments following the circumference of the nanotube, thus perpendicular to the current flow whereas the remaining lie at intermediate orientations between the parallel and antiparallel to the NTs axis. Following the same argument, we attribute the reduction in the resistance curve around *H*_*c*_ in the IP configuration (Fig. 5c) to the creation of vortex domain walls, as previously suggested from AMR experiments performed on an individual Ni nanotube^42^. The differences between the magneto-transport properties of CNW and CNT networks can be better understood from the AMR curves recorded in the OOP direction (see Fig. 6), as they display more evident changes. The AMR curves measured in the OOP direction for CNW networks with NW diameters of 105 nm and 230 nm, shown in Fig. 6, both display a decrease in resistivity near zero field. This feature results from the misalignment between the magnetization and the electrical current, which is consistent with the reduced remanence magnetization at zero field (see Fig. 3 for the CNW network) in view of the large diameter of the NWs. However, it is interesting to note that the AMR curve minimum for both CNW networks does not coincide with the resistance at coercivity (shown as lozenges), as seen in Fig. 6. Indeed, starting from the saturated positive state, the resistance minimum for CNWs is observed at positive field values, in contrast to what is observed in arrays of parallel nanowires with only magnetostatic contribution to the magnetic anisotropy. In that case, the resistance of the NWs reaches the minimum values when the direction of the magnetic field is reversed and coincide with the coercive field^35,39–41,46^. This effect can be ascribed to the interconnected NW architecture and the presence of domain walls that are formed at the magnetic junctions once the external field is reduced, thus giving rise at low field to complex spin configurations formed by a series of domains in the axially saturate states separated by domains walls at the segment intersections. Comparing both OOP AMR curves, it is clear that the larger the NWs diameter the more pronounced the decrease in resistivity in the vicinity of zero field. The decrease in the depth of the AMR curve minimum for lower NW diameters means that the nucleation of domain walls, where locally the moments are perpendicular to the current, extends over a smaller volume at the intersection of the NW segments. As may be expected, the junction volume has distinct effects on the domain wall configuration and the initial magnetic switching. In our case, *H*_*c*_ decreases with the wire diameter, as larger diameter is more likely to form multiple domains at the intersection of the NW segments, due to the demagnetization effect.

## Conclusion

We explored the fabrication and the corresponding magnetic and magneto-transport behavior of a complex nano-architecture based on crossed nanotube networks. Combining the use of the electrochemical dealloying method along with track etched PC membranes with interconnected cylindrical nanopores has lead to cost-effective and large-scale CNT networks with a high control over structural and magnetic properties. These features have been carefully modified via the control over the reduction or deposition potential of crossed core(Cu)/shell(Ni) nanocables, leading to CNT networks with different wall thickness after carrying out the dealloying step of the Cu core. An expression based on simple phenomenological arguments that relates the wall thickness and the diameter of the nanotubes has been proposed and validated. This expression has been proved to be valid also for nanotubes other than CNT networks fabricated by the electrochemical dealloying technique. Besides, in contrast to other fabrication techniques like ALD and CVD, the ability of fabricating both large-scale interconnected CNT and CNW networks by simple electrochemical methods highlights the possibility of modifying both the lineshape of the AMR curves and the overall magnetoresistive behavior. Particularly, CNT networks display a magnetoresistive behavior that is dominated by a vortex reversal mechanism that leads to a sharp fall in electrical resistivity at negative reversal fields. Conversely, CNW networks present an unusual magnetoresistive behavior dominated by the nucleation of domain walls at the crossing zones of the NWs, which leads to minima in resistivity at positive reversal fields, contrary to the usual behavior previously reported in parallel NW arrays. The present results are of special interest for the development of potential device applications with a wide range of magnetic and magneto-transport behavior for their use as magnetic sensors with 3-D resolution.

## Methods

### Synthesis of 3-D CNT and CNW networks

Nickel CNT and CNW networks have been grown by a standard three-probe electrodeposition technique into the interconnected pores of nanoporous polycarbonate (PC) templates, obtained by performing a sequential two-step exposure of energetic heavy ions, similar to those described elsewhere^14,15,47^. As explained previously, 22 *μ*m thick porous PC membranes with interconnected and crossed nanopores have been used for the electrodeposition of CNT networks. The interconnected pore architecture is achieved by exposing a PC film to a sequential irradiation process (see Fig. 1a,b). By following a previously reported protocol^23^, the latent tracks were chemically etched in a 0.5 M NaOH aqueous solution at 70 °C at different etching times. As a result two different templates with 105 nm or 230 nm diameter nanopores (*d*) and constant volumetric porosity (*P*) of about 20%, were designed. Following this procedure, a Cr(10 nm)/Au(2*μ*m) layer has been evaporated onto one side of the membranes to serve as a cathode for the electrodeposition, which also served as ground plane for the ferromagnetic resonance (FMR) experiments and as electrode for the magnetotransport measurements. Ni CNTs (*ϕ* = 230 nm) were fabricated following the dealloying process previously reported for nanowire parallel arrays^22,26,27^, where core-shell Ni/Cu nanocables were grown at a constant deposition potential in the range from −0.8 V to −1.1 V, followed by the selective electrochemical etching of the Cu-rich core at an oxidation potential of +0.2 V. Ni CNWs were grown at a constant deposition potential of −1 V. For the growth of CNTs and CNWs, the following electrolytes were respectively used: 0.4 M Ni(H_2_NSO_3_)_2_·4H_2_O + 0.05 M CuSO_4_·5H_2_O + 0.1 M H_3_BO_3_ and 1 M NiSO_4_·6H_2_O + 0.5 M H_3_BO_3_, with the pH adjusted to 4.

### Structural characterization and EDX analysis

The PC template was removed using dichloromethane in order to characterize the morphology of the CNT and CNW networks using a field-emission scanning electron microscope (FE-SEM), JEOL 7600F. Energy dispersive X-ray (EDX) analysis was carried out, using the JED-2300 AnalysisStation of this microscope, on Ni CNT and Cu/Ni core/shell CNW networks. At least 10 EDX mappings at a magnification of X30,000 and acceleration voltage of 15 kV were carried out on each sample and the ZAF Method Standarless Quantitative Analysis was used to quantify the amount of atomic % of Cu and Ni.

### Magnetic characterization by AGM and FMR

An alternating gradient field magnetometer (AGFM-Lakeshore) with a maximum applied field of ±10 kOe has been used to record magnetization curves at room temperature with the external field applied along the in-plane (IP) and out-of-plane (OOP) directions of the CNW network film. Ferromagnetic resonance (FMR) measurements were performed at room temperature at a constant frequency in the range of 100 MHz to 50 GHz, by sweeping the magnetic field applied in the OOP direction from 10 kOe down to zero field. These measurements were done in the microstrip line wave-guide configuration^15^, which is based on a 500 *μ*m wide and 500 nm thick metallic microstrip evaporated on the free side of the PC membrane after electrodeposition. For this configuration, overfilling of the porous templates with either CNWs or CNTs were not allowed in order to avoid electrical contact between the ground plane and the microstrip line.

### Electrical magneto-transport characterization

Magnetoresistance (MR) measurements were carried out by using an experimental setup based on a two-probe measurement system, while sweeping a magnetic field between ±10 kOe. The magneto-transport measurement system consists in a similar configuration as the microstrip line for the FMR measurements, however, in this case overfilling the porous templates with CNWs and CNTs were allowed in order to have a good electrical contact between the ground plane and the microstrip line. In this configuration, the current is directly injected to the branched CNW structure from the ground plane to the microstrip line, thanks to the high degree of electrical connectivity of the networks. The electrical contacts were made using Ag paint. For each sample, the input power was kept below 0.1 *μ*W to avoid self-heating, and the resistance was measured within its ohmic resistance range with a resolution of one part in 10^5^.

## Electronic supplementary material

Supplementary Information

## References

[1] Sander D (2017). The 2017 magnetism roadmap. J. Phys. D: Appl. Phys..

[2] Yershov KV, Kravchuk VP, Sheka DD, Gaididei Y (2016). Curvature and torsion effects in spin-current driven domain wall motion. Phys. Rev. B.

[3] Fernández-Pacheco A (2017). Three-dimensional nanomagnetism. Nat. Commun..

[4] Parkin SSP, Hayashi M, Thomas L (2008). Magnetic domain-wall racetrack memory. Sci..

[5] Parkin S, Yang S-H (2015). Memory on the racetrack. Nat. Nanotechnol..

[6] Melzer M (2015). Imperceptible magnetoelectronics. Nat. Commun..

[7] Makarov D, Melzer M, Karnaushenko D, Schmidt OG (2016). Shapeable magnetoelectronics. Appl. Phys. Rev..

[8] Li H (2016). Stretchable spin valve with stable magnetic field sensitivity by ribbon-patterned periodic wrinkles. ACS Nano.

[9] Chen J-Y, Lau Y-C, Coey JMD, Li M, Wang J-P (2017). High performance MgO-barrier magnetic tunnel junctions for flexible and wearable spintronic applications. Sci. Reports.

[10] Wang Z (2016). Highly sensitive flexible magnetic sensor based on anisotropic magnetoresistance effect. Adv. Mater..

[11] Cardoso S (2017). Challenges and trends in magnetic sensor integration with microfluidics for biomedical applications. J. Phys. D: Appl. Phys..

[12] Chang C-H, Ortix C (2017). Theoretical prediction of a giant anisotropic magnetoresistance in carbon nanoscrolls. Nano Lett..

[13] Yi-Wei L, Qing-Feng Z, Run-Wei L (2013). Fabrication, properties, and applications of flexible magnetic films. Chin. Phys. B.

[14] Araujo E (2015). Artificially modified magnetic anisotropy in interconnected nanowire networks. Nanoscale.

[15] da Câmara Santa Clara Gomes T (2016). Interplay between the magnetic and magneto-transport properties of 3D interconnected nanowire networks. J. Appl. Phys..

[16] da Câmara Santa Clara Gomes T (2017). 3-D interconnected magnetic nanofiber networks with multifunctional properties. IEEE Transactions on Magn..

[17] Hrkac G, Dean J, Allwood DA (2011). Nanowire spintronics for storage class memories and logic. Philos. Transactions Royal Soc. Lond. A: Math. Phys. Eng. Sci..

[18] Escrig J, Daub M, Landeros P, Nielsch K, Altbir D (2007). Angular dependence of coercivity in magnetic nanotubes. Nanotechnol..

[19] Allende S, Escrig J, Altbir D, Salcedo E, Bahiana M (2008). Angular dependence of the transverse and vortex modes in magnetic nanotubes. The Eur. Phys. J. B.

[20] Albrecht O (2011). Experimental evidence for an angular dependent transition of magnetization reversal modes in magnetic nanotubes. J. Appl. Phys..

[21] Proenca MP (2013). Magnetic interactions and reversal mechanisms in co nanowire and nanotube arrays. J. Appl. Phys..

[22] Velázquez-Galván Y (2014). Dipolar interaction in arrays of magnetic nanotubes. J. Physics: Condens. Matter.

[23] Ferain E, Legras R (2003). Track-etch templates designed for micro- and nanofabrication. Nucl. Instruments Methods Phys. Res. Sect. B: Beam Interactions with Mater. Atoms.

[24] de la Torre Medina J, Darques M, Piraux L (2008). Strong low temperature magnetoelastic effects in template grown ni nanowires. J. Phys. D: Appl. Phys.

[25] Piraux L, Antohe V-A, Ferain E, Lahem D (2016). Self-supported three-dimensionally interconnected polypyrrole nanotubes and nanowires for highly sensitive chemiresistive gas sensing. RSC Adv..

[26] Wang Q, Wang G, Han X, Wang X, Hou JG (2005). Controllable template synthesis of ni/cu nanocable and ni nanotube arrays: A one-step coelectrodeposition and electrochemical etching method. The J. Phys. Chem. B.

[27] Liu Z (2008). Exploiting finite size effects in a novel core/shell microstructure. J. Appl. Phys..

[28] Antohe V-A, Nysten E, Martinez-Huerta JM, Pereira de Sa PM, Piraux L (2017). Annealing effects on the magnetic properties of highly-packed vertically-aligned nickel nanotubes. RSC Adv..

[29] Wang C (2005). Thermodynamic database of the phase diagrams in copper base alloy systems. J. Phys. Chem. Solids.

[30] Stoner EC, S. FR, Wohlfarth EP (1948). A mechanism of magnetic hysteresis in heterogeneous alloys. Philos. Transactions Royal Soc. Lond. A: Math. Phys. Eng. Sci.

[31] Liu Z, Elbert D, Chien C-L, Searson PC (2008). FIB/TEM characterization of the composition and structure of core/shell Cu-Ni nanowires. Nano Lett..

[32] Martínez-Huerta JM, de la Torre Medina J, Piraux L, Encinas A (2012). Self consistent measurement and removal of the dipolar interaction field in magnetic particle assemblies and the determination of their intrinsic switching field distribution. J. Appl. Phys..

[33] Ferré R, Ounadjela K, George JM, Piraux L, Dubois S (1997). Magnetization processes in nickel and cobalt electrodeposited nanowires. Phys. Rev. B.

[34] Wernsdorfer W (1996). Nucleation of magnetization reversal in individual nanosized nickel wires. Phys. Rev. Lett..

[35] Pignard S (2000). Study of the magnetization reversal in individual nickel nanowires. J. Appl. Phys..

[36] Paulus P, Luis F, Kröll M, Schmid G, de Jongh L (2001). Low-temperature study of the magnetization reversal and magnetic anisotropy of Fe, Ni, and Co nanowires. J. Magn. Magn. Mater..

[37] Chen Z (2012). The magnetic y-branch nanojunction: Domain-wall structure and magneto-resistance. Appl. Phys. Lett..

[38] Encinas-Oropesa A, Demand M, Piraux L, Huynen I, Ebels U (2001). Dipolar interactions in arrays of nickel nanowires studied by ferromagnetic resonance. Phys. Rev. B.

[39] Wegrowe J-E, Kelly D, Franck A, Gilbert SE, Ansermet J-P (1999). Magnetoresistance of ferromagnetic nanowires. Phys. Rev. Lett..

[40] Ohgai T (2003). Template synthesis and magnetoresistance property of Ni and Co single nanowires electrodeposited into nanopores with a wide range of aspect ratios. J. Phys. D: Appl. Phys..

[41] Wong DW (2016). Current-induced three-dimensional domain wall propagation in cylindrical NiFe nanowires. J. Appl. Phys..

[42] Ruffer D (2012). Magnetic states of an individual ni nanotube probed by anisotropic magnetoresistance. Nanoscale.

[43] Bordignon G (2007). Analysis of magnetoresistance in arrays of connected nano-rings. IEEE Transactions on Magn..

[44] Zhu FQ, Fan DL, Cammarata RC, Chien CL (2004). Magnetic and magneto-transport properties of electrodeposited magnetic nano-network on laser modified Au surface. J. Appl. Phys..

[45] McGuire T, Potter R (1975). Anisotropic magnetoresistance in ferromagnetic 3d alloys. IEEE Transactions on Magn..

[46] Jaccard Y, Guittienne P, Kelly D, Wegrowe J-E, Ansermet J-P (2000). Uniform magnetization rotation in single ferromagnetic nanowires. Phys. Rev. B.

[47] Rauber M (2011). Highly-ordered supportless three-dimensional nanowire networks with tunable complexity and interwire connectivity for device integration. Nano Lett..

