# Supplementary material for: Large-scale 3-D interconnected Ni nanotube networks with controlled structural and magnetic properties
Source: Sci Rep. 2018 Sep 28;8:14555. doi: 10.1038/s41598-018-32437-8 (PMC6162309; doi:10.1038/s41598-018-32437-8)
Supplement: Supplementary file 1 — Supplementary Information [file 41598_2018_32437_MOESM1_ESM.pdf]

Supplementary Information

Large-scale 3-D interconnected Ni nanotube networks with controlled structural and magnetic properties

Joaquín de la Torre Medina[1,*,+], Tristan da Câmara Santa Clara Gomes[2], Yenni G. Velázquez Galván[2], and Luc Piraux[2]

[1]Instituto de Investigaciones en Materiales / Unidad Morelia, Universidad Nacional Autónoma de México, Morelia, Mexico.
[2]Institute of Condensed Matter and Nanosciences, Université catholique de Louvain, Louvain-la-Neuve, Belgium.
[*]delatorre@iim.unam.mx
[+]Part of the work has been done at Institute of Condensed Matter and Nanosciences - UCL.

The following sections describe the procedures to obtain the analytical expressions used for the ferromagnetic resonance and anisotropic magnetoresistance experiments.

Ferromagnetic resonance condition for CNT and CNW networks

According to the Smit and Beljers formalism, the classical ferromagnetic resonance (FMR) condition is given by[1]

$$\left(\frac{f_r}{\gamma}\right)^2 = [H_{eff}\cos 2\theta_0 + H_r\cos(\theta_0-\theta_H)] \times \left[H_{eff}\cos^2\theta_0 + H_r\cos(\theta_0-\theta_H)\right], \quad (1)$$

where $\gamma = 3.09$ GHz/kOe is the gyromagnetic ratio for Ni, H_r is the resonance field, H_{eff} is the effective anisotropy field of the network and θ_H and θ_0 are respectively the angle of the saturated magnetization and the angle of the applied field measured with respect to the OOP direction. In both CNT and CNW networks, the magnetization is oriented parallel to the applied field in the OOP direction and tilted to a fixed angle θ_0 with respect to their axis. In the saturated state ($\theta_0 = \theta_H$) with the external field applied in the OOP direction, the FMR condition of equation (1) then writes

$$f_r = \gamma\sqrt{H_r^2 + A_1 H_{eff} H_r + A_2 H_{eff}^2}. \quad (2)$$

In this equation the constants A_1 and A_2 depend on the exact θ_0 value, so

$$A_1 = \cos 2\theta_0 + \cos^2\theta_0 \quad (3)$$

and

$$A_2 = \cos 2\theta_0 \cos^2\theta_0. \quad (4)$$

Considering an orientation angle $\theta_0 = 25°$ for both CNTs and CNWs, these constants are $A_1 = 1.464$ and $A_2 = 0.528$. For the case where only MS contributions take place, the effective anisotropy field of a particular network is proportional to the saturation magnetization (M_s). The topological differences of the distinct CNW and CNT networks can be extracted by comparing their anisotropy field H_{eff} with respect to the anisotropy field $H_F = 4\pi M_s$ for the infinite magnetic thin film, which can be considered as a reference system for planar interconnected networks. Indeed, by defining a magnetostatic factor N_M as the ratio H_{eff}/H_F ensures that it reproduces only the topological aspects of purely magnetostatic planar networks, as it is independent on M_s. Thus, normalizing the resonance condition of equation (2) by the field H_F permits to identify precise differences in the topology of CNW and CNT networks grown under different conditions. By defining the normalized resonance frequency and resonance field respectively by $f_N = f_r/(\gamma H_F)$ and $h_N = H_r/H_F$, the resonance condition of equation (2) reads

$$f_N = \sqrt{h_N^2 + A_1 N_M h_N + A_2 N_M^2}. \quad (5)$$

This equation can be used along with the experimental data to obtain N_M as fitting parameter, which in turn allows determining the effective field $H_{eff} = 4\pi M_s N_M$.

Anisotropic magnetoresistance for CNT and CNW networks

According to the AMR relation[2], the electrical resistivity of magnetized materials depends on the relative orientation (θ) between the electrical current along the NWs (or NTs) and the magnetization, that is

$$\rho(\theta) = \rho_\perp + (\rho_{||} - \rho_\perp)\cos^2\theta. \tag{6}$$

where $\rho_{||}$ ($\rho_\perp$) is the resistivity of the CNW network in the high (low) resistance state when the local magnetization and current paths are parallel (perpendicular) to each other. At the saturated state along the OOP or IP direction, the angle between the magnetization and electrical current is $\theta = \theta_0$ or $\theta = \frac{\pi}{2} - \theta_0$, with θ_0 being the constant angle between the NWs or NTs axis and the normal direction to the porous template. Evaluating equation (6) at these angles yields the resistivities at saturation ρ_{oop} and ρ_{ip} along the OOP and IP directions, which can be combined in order to obtain the low resistance state, that is

$$\rho_\perp = \frac{k\rho_{ip} - (1-k)\rho_{oop}}{2k-1}, \tag{7}$$

where $\rho_{oop} = k\rho_{||} + (1-k)\rho_\perp$ and $\rho_{ip} = (1-k)\rho_{||} + k\rho_\perp$, with $k = \cos^2\theta_0$. Particularly, $k = 0.8214$ for the CNT and CNW networks studied in this work for which $\theta_0 = 25°$. Further, combining equation (7) with the resistance states at saturation allows determining the high resistance state ($\rho_{||}$) via the following expression

$$\rho_{||} = \rho_{ip} + \rho_{oop} - \rho_\perp. \tag{8}$$

With the knowledge of the low and high resistance states, the AMR ratio can be then obtained as

$$\frac{\Delta\rho}{\rho} = \frac{\rho_{||} - \rho_\perp}{\rho_{av}}. \tag{9}$$

where $\rho_{av} = \frac{1}{3}\rho_{||} + \frac{2}{3}\rho_\perp$ is the average magnetoresistance in 3D systems. Equations (7), (8) and (9) combined together allow obtaining accurate AMR ratios for Ni CNW and CNT networks.

References

1. Encinas-Oropesa, A., Demand, M., Piraux, L., Huynen, I. & Ebels, U. Dipolar interactions in arrays of nickel nanowires studied by ferromagnetic resonance. *Phys. Rev. B* **63**, 104415 (2001).
2. McGuire, T. & Potter, R. Anisotropic magnetoresistance in ferromagnetic 3d alloys. *IEEE Transactions on Magn.* **11**, 1018–1038 (1975).
